# AAV encoding miRNAs as a new therapeutic for choroidal neovascularization

**DOI:** 10.1016/j.omtn.2026.103135

**Published:** 2026-10-27

**Authors:** Marc-Antoine Turcotte, Carole Dinant, Ian M. MacDonald

**Affiliations:** 1 Département de Chirurgie, Division d’ophtalmologie, Centre de recherche du Centre hospitalier universitaire Sainte-Justine, Montréal, Québec, H3T 1C5, Canada; 2 Département d’ophtalmologie, Faculté de médecine, Université de Montréal, Montréal, Québec, H3T 1P1, Canada

## Main text

Better treatments are urgently needed for eye conditions when existing therapies fall short. Age-related macular degeneration (AMD) is a significant contributor to irreversible central vision impairment in an aging population. The neovascular form of AMD (nAMD) accounts for most severe vision loss due to choroidal neovascularization (CNV), a process often leading to subretinal hemorrhage, photoreceptor cell death, and ultimately permanent fibrovascular scarring.^1^ At present, standard treatments use recombinant anti-VEGF fusion proteins or antibodies injected intravitreally every few months.^2^ Despite their clinical benefit, these treatments are not a definitive cure; they provide limited improvement in a subset of nAMD patients, and their repeated administration adds considerable treatment and financial burden. Developing new therapeutic strategies for nAMD remains a pressing clinical need.

The study by Han et al.^3^ in the December issue of *Molecular Therapy Nucleic Acids* demonstrated an elegant therapeutic strategy, where the utilization of adeno-associated virus (AAV) vectors coding for microRNAs (miRNAs) against MAPK-related genes led to a therapeutic improvement in a laser-induced CNV mouse model. Briefly, the investigators designed two different treatments, one containing three AAV constructs each coding for a different miRNA targeting MAPK-related genes (referred to as the mixture), and the other, an AAV construct coding for the same three miRNAs (referred to as the tandem). The transduction of both treatments in an immortalized human retinal pigment epithelial (RPE) cell line led to a reduction of H_2_O_2_ stress-induced apoptosis. This observation was associated with the reduction of the p-JNK/JNK, p-p38/p38, and p-ERK/ERK protein ratios. Furthermore, these findings were confirmed in a laser-induced CNV mouse model following intravitreal injection. Mice that were given the treatment displayed reduced CNV leakage, reduced structural lesions observed by optical coherence tomography, and an anti-angiogenic effect. Lastly, RPE morphology restoration, epithelial-mesenchymal transition suppression, and fibrosis suppression in CNV lesion sites were observed seven days after laser photocoagulation. Although these findings are important for the AMD community and could provide hope for patients, a few caveats need to be thoroughly investigated, including the phenotypic difference between both vectors, their advantage over current treatments, side effects, and waning therapeutic efficacy.

The findings clearly showed an improvement in efficacy for the mixture AAV compared to the tandem construct. A few questions arise from these observations. (1) Did this difference result from different quantities of miRNAs transcribed between both treatments or from differences in transduction efficiency? In the transduced RPE cell line, a 2-fold decrease in each miRNA was observed. Moreover, transduction efficiency was only evaluated with the mixture therapeutic. (2) In the tandem treatment, would either increasing the AAV quantity, optimizing the promoters and distances between each miRNA or duplicating the U6-shRNA-terminator locus lead to effects similar to the mixture treatment? From a pharmaceutical standpoint, the single tandem AAV simplifies bioprocessing and purification relative to the mixture treatment, which relies on three independent AAV vectors. 3) Would the combination of two or only one miRNA lead to a significant therapeutic effect? Although the AAVs containing individual miRNA have been tested in ARPE-19 cells, their effects have not been investigated *in vivo*. The reduction of the number of miRNAs would be useful to reduce potential non-targeted effects and side effects of the treatment.

The comparison of these new AAVs with current treatments is important to establish the merits and limitations of each therapy. The authors concentrated their effort on comparing the treatment with aflibercept, a recombinant anti-VEGF fusion protein.^2^ Aflibercept treatment led to higher p-ERK, p-JNK, and p-p38 ratios compared to the mixture of AAVs (although not significant for p-ERK). This indicates an overactive MAPK pathway compared to the AAVs. However, these results should be approached with caution. While they may seem like an improvement, there are no clear guidelines on how to compare quantities of fusion proteins and AAVs. Furthermore, only one concentration of AAVs and aflibercept was reported in the paper (40 μg/μL for aflibercept versus 10^9^ genomic copies for the AAVs). Thus, either a higher concentration of aflibercept or a lower concentration of AAVs could equalize these three protein ratios between both treatments. The same applies to all *in vivo* observations. Additionally, the authors did not contextualize their findings to current phase 2 and 3 AAV-based clinical candidates, such as RGX-314, ADVM-022, and 4D-150, which transduce retinal cells to express anti-VEGF proteins and/or microRNAs against VEGF.^4^ In clinical trials, these benchmark therapies have demonstrated durable therapeutic expression lasting at least 24 weeks alongside manageable, mild-to-moderate intraocular inflammation that is generally responsive to corticosteroid interventions.

Another clinical aspect that remains uninvestigated is the variability in response to anti-VEGF therapy. Approximately 10%–20% of patients with nAMD show a poor or incomplete response, with some showing persistent disease activity or limited visual recovery despite treatment.^5^ It would therefore be interesting to investigate whether similar variability in response could occur with the authors’ new treatments, and whether specific patient subgroups may respond differently. The mechanisms underlying incomplete response to anti-VEGF therapy are likely multifactorial and may involve neovascular remodeling, inflammation, and fibrosis.^5^

Both subretinal and intravitreal AAV injections trigger ocular inflammation via innate and adaptive immune recognition of the capsid and genome.^6^ Although the authors mentioned that the MAPK pathway is known to promote inflammation, inflammation was not investigated in this study. Furthermore, mice were sacrificed 12 days following the injection, which limits the long-term study of ocular inflammation. Inflammation following AAV administration can be influenced by several factors, including high AAV doses, the presence of empty capsids, the viral genome or transgene, pre-existing immunity, and the route of administration.^7^ Considering that the AAV2.7m8 engineered capsid has been reported in clinical trials to cause inflammation, there is a high probability that it will occur with this therapy.^8^ The presence of the three miRNAs could further accentuate or diminish this phenotype. Thus, we believe that optimizing multiple components (capsid, genome organization, and miRNA quantity) is essential for this treatment to reach high efficacy, particularly considering the low incidence of intraocular inflammation with standard treatments.^2^

As the authors pointed out in their discussion, a long-term investigation will be needed to monitor the progression of the disease, the amount of miRNA expressed, the levels of MAPK-related genes, and, as previously discussed, inflammation. The efficacy of the treatment will also need to be shown in nAMD. Laser-induced CNV is indeed an interesting model for nAMD, but it only mimics an acute phenotype of the disease that does not account for the chronic and progressive development of nAMD and its genetic architecture.^9^ It will be important to determine whether these effects are maintained in a chronic model and over longer follow-up. The need for a durable effect is also particularly relevant for AAV-based therapies, as repeat administration of the same vector may be limited by anti-AAV immune responses.^7^

In summary, the therapy presented by the authors, based on AAV and miRNA, is an elegant approach to treat nAMD. A few questions remain, particularly the efficacy compared to the current therapies, the need for capsid and genome bioengineering, the long-term safety profile, and the potential variability in treatment response. We are enthusiastic to see future studies addressing our comments.

## Declaration of interests

The authors declare no competing interests.
